# Regulation of muscle atrophy-related genes by the opposing transcriptional activities of ZEB1/CtBP and FOXO3

**DOI:** 10.1093/nar/gky835

**Published:** 2018-10-10

**Authors:** Chiara Ninfali, Laura Siles, Douglas S Darling, Antonio Postigo

**Affiliations:** 1 Group of Transcriptional Regulation of Gene Expression, Department of Oncology and Hematology, IDIBAPS, Barcelona 08036, Spain; 2 Center for Genetics and Molecular Medicine and Department of Immunology and Infectious Diseases, University of Louisville, Louisville, KY 40202, USA; 3 Molecular Targets Program, James G. Brown Cancer Center, Louisville, KY 40202, USA; 4 ICREA, Barcelona 08010, Spain

## Abstract

Multiple physiopathological and clinical conditions trigger skeletal muscle atrophy through the induction of a group of proteins (atrogenes) that includes components of the ubiquitin–proteasome and autophagy-lysosomal systems. Atrogenes are induced by FOXO transcription factors, but their regulation is still not fully understood. Here, we showed that the transcription factor ZEB1, best known for promoting tumor progression, inhibits muscle atrophy and atrogene expression by antagonizing FOXO3-mediated induction of atrogenes. Compared to wild-type counterparts, hindlimb immobilization in *Zeb1*-deficient mice resulted in enhanced muscle atrophy and higher expression of a number of atrogenes, including Atrogin-1/*Fbxo32*, MuRF1/*Trim63, Ctsl, 4ebp1, Gabarapl1, Psma1* and *Nrf2*. Likewise, in the C2C12 myogenic cell model, ZEB1 knockdown augmented both myotube diameter reduction and atrogene upregulation in response to nutrient deprivation. Mechanistically, ZEB1 directly represses *in vitro* and *in vivo Fbxo32* and *Trim63* promoter transcription in a stage-dependent manner and in a reverse pattern with MYOD1. ZEB1 bound to the *Fbxo32* promoter in undifferentiated myoblasts and atrophic myotubes, but not in non-atrophic myotubes, where it is displaced by MYOD1. ZEB1 repressed both promoters through CtBP-mediated inhibition of FOXO3 transcriptional activity. These results set ZEB1 as a new target in therapeutic approaches to clinical conditions causing muscle mass loss.

## INTRODUCTION

Under homeostatic conditions, skeletal muscle maintains a balance between protein synthesis and proteolysis by finely tuning hypertrophic and atrophic signals (reviewed in 1-3). Multiple physiopathological and clinical conditions (e.g. immobilization, aging, denervation) result in skeletal muscle atrophy, a reduction in muscle mass and in the cross-sectional area (CSA) of myofibers.

Ultimately, muscle atrophy is mediated by a number of genes collectively referred as ‘atrogenes’ and that includes members of the ubiquitin–proteasome and the autophagy-lysosomal systems (4–9). Most sarcomeric proteins are degraded by the ubiquitin–proteasome pathway; E3 ubiquitin ligases bind to their substrates and catalyze the transfer of ubiquitin from the E2 enzyme targeting proteins for subsequent degradation by the 26S proteasome (10,11). In turn, organelles, particularly mitochondria, are degraded by proteasomal degradation and autophagy (12,13). The two archetypal atrogene proteins whose expression increases the strongest during muscle atrophy are the E3 ubiquitin ligases Atrogin-1 (also known as MAFbx and encoded by the gene *Fbxo32*) and MuRF1 (encoded by *Trim63*) (4). *Fbxo32* (-/-) and *Trim63* (-/-) mice exhibit reduced muscle sparing in response to atrophy-inducing experimental protocols (4). Atrogin-1 and MuRF1 expression is directly activated by O-type forkhead transcription factors (FOXO), chiefly by FOXO3 (2,9,14). FOXO3 also activates atrogenes involved in the autophagy-dependent clearance of organelles (9,12,13). Nevertheless, the transcriptional mechanisms regulating the expression of Atrogin-1, MuRF1 and other atrogenes are not completely understood. Surprisingly, we found here that the transcription factor ZEB1 inhibits atrogene expression and muscle atrophy in a stage-dependent manner through repression of FOXO3 transcriptional activity.

Although ZEB1 is best known for promoting tumor progression by triggering an epithelial-to-mesenchymal transition (EMT) in cancer cells (15–17), it also plays important roles in embryogenesis—*Zeb1* (-/-) mice die before birth—and cell differentiation (18,19). ZEB1 is expressed in the primary myotome, where the first muscle progenitors arise (18), and imposes a stage-dependent inhibition of muscle differentiation, so *Zeb1* (-/-) and *Zeb1* (+/-) embryos display premature expression of adult muscle differentiation genes (20,21). Both mutation and overexpression of ZEB1’s ortholog in *Drosophila* (zfh-1) disrupt somatic musculature (21,22). However, the expression and role of ZEB1 in muscle atrophy have not been explored. ZEB1 is induced by multiple signaling pathways whose activity and gene targets it modulates positively or negatively by recruitment of transcriptional co-activators (e.g. p300) or co-repressors (e.g. CtBP) (15,16,23–27).

Here, we showed that, compared to wild-type counterparts, hindlimb immobilization in *Zeb1* (+/-) mice resulted in enhanced muscle atrophy and higher expression of a number of atrogenes, including *Fbxo32, Trim63, Ctsl, 4ebp1, Gabarapl1, Psma1* and *Nrf2*. Likewise, in the C2C12 myogenic cell model, ZEB1 knockdown amplified both myotube diameter reduction and atrogene upregulation in response to nutrient deprivation. We identified ZEB1-binding sites in the regulatory regions of *Fbxo32* and *Trim63* and confirmed its direct binding and repression of these promoters in a stage-dependent manner and in a reverse pattern with MYOD1. ZEB1 bound to the *Fbxo32* promoter in undifferentiated myoblasts and atrophic myotubes, but not in non-atrophic myotubes where it is displaced by MYOD1. ZEB1-dependent repression of the *Fbxo32* promoter in atrophic muscles was also validated *in vivo* by bioluminescence imaging. Mechanistically, ZEB1 repressed atrogene expression through CtBP-dependent inhibition of the transcriptional activity of FOXO3.

The data presented here indicate that ZEB1 limits unrestrained muscle atrophy and atrogene overexpression in response to atrophic-inducing stimuli, thus offering a new target in therapeutic approaches to physiopathological and clinical conditions dealing with muscle mass loss.

## MATERIALS AND METHODS

### Mouse samples

The use of mouse models in this study was approved by Animal Experimentation Ethics Committee of the University of Barcelona under protocol number DAAM 8563. The source of mouse models used in the study, the hindlimb immobilization protocol and the *in vivo* analysis of atrogene promoter activity are detailed in Supplementary Data.

### Cell lines and cell culture

C2C12 and 293T cell lines were obtained from the American Type Culture Collection (ATCC)-LGC Standards (Middlesex, England, UK). The culture conditions for myotube differentiation and starvation are detailed in Supplementary Data.

### Antibodies, and DNA and RNA oligonucleotides

The antibodies used in western blot, and in the immunostaining of mouse muscle samples and C2C12 myotubes are detailed in Supplementary Data. DNA oligonucleotides used as primers in quantitative real-time polymerase chain reaction (qRT-PCR) are listed in the Supplementary Data. Lastly, RNA oligonucleotides used in RNA interference are described in the Supplementary Data.

### Gene and protein expression

RNA extraction and subsequent analysis of gene expression by qRT-PCR, and transcriptional studies by luciferase reporter assays are described in Supplementary Data. Analysis of protein expression in mouse tissue samples and C2C12 myotubes as well as myofibers’ CSA analysis of muscle sections are described in Supplementary Data.

### Statistical analysis

Statistical analysis of data shown was performed using GraphPad Prism for Mac version 5.0a (GraphPad Software Inc., La Jolla, CA, USA). Normal distribution of the data was determined with Kolmogorov–Smirnov test. Statistical significance of the normally distributed data was assessed with a *t*-test and with a non-parametric Mann–Whitney *U* test for those with non-normal distribution. Error bars in histograms represent standard errors of means. Relevant comparisons were labeled as either significant at the *P* ≤ 0.001 (***), *P* ≤ 0.01 (**) or *P* ≤ 0.05 (*) levels, or non-significant (ns) for values of *P* > 0.05.

## RESULTS

### ZEB1 protects skeletal muscle from sparing upon immobilization

**Figure 1.**
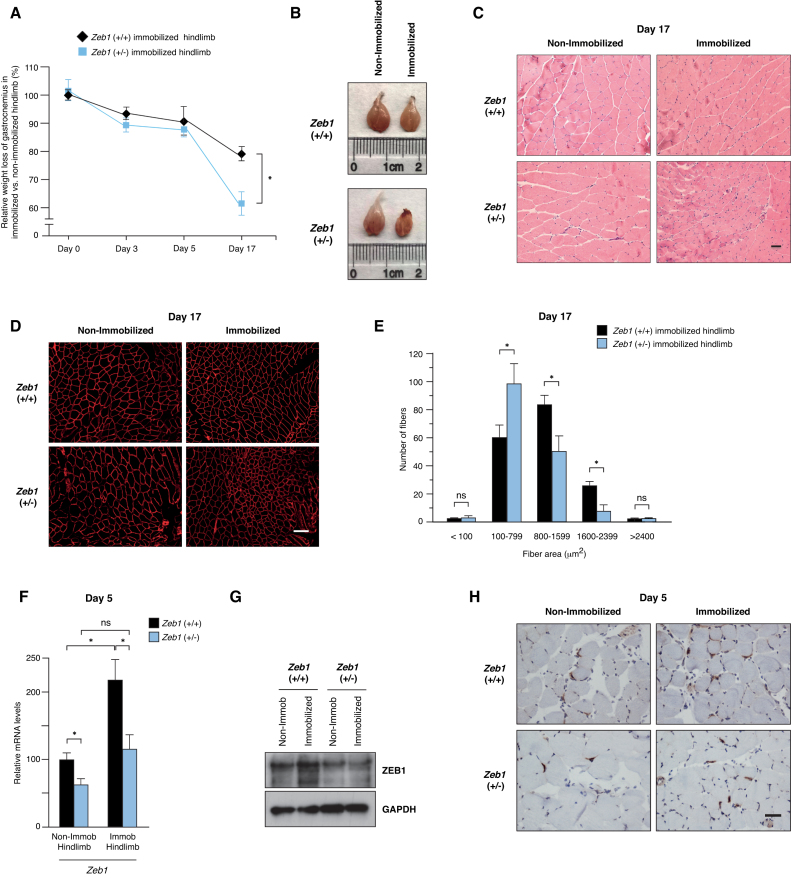
ZEB1 protects skeletal muscle from sparing upon immobilization. (**A**) Two-to-three-month old wild-type and *Zeb1* (+/-) mice were subjected to unilateral hindlimb immobilization for different periods as described in Supplementary Data. At each time point, mice were euthanized and the weight of their immobilized gastrocnemius muscles was assessed with respect to that in the contralateral non-immobilized hindlimb. The weight of the gastrocnemius in the immobilized hindlimb *vis-à-vis* that in the non-immobilized at the start of the protocol (day 0) was set arbitrarily to 100. At least five mice of each genotype were examined. (**B**) As in (A), representative images of non-immobilized and immobilized gastrocnemius muscles from wild-type and *Zeb1* (+/-) mice at day 17 of the immobilization protocol. (**C**) Wild-type and *Zeb1* (+/-) mice were subjected to unilateral hindlimb immobilization during 17 days as in (A), euthanized and their gastrocnemius muscles stained for hematoxylin/eosin. Scale bar: 50 μm. (**D**) As in (C), but sections were stained with an antibody against laminin (clone 48H-2). Scale bar: 100 μm. (**E**) Myofiber cross-sectional analysis in the immobilized gastrocnemius of wild-type and *Zeb1* (+/-) mice at day 17 of the immobilization protocol. Myofiber area was assessed as described in Supplementary Data. A total of 160 myofibers were measured from at least eight mice, half from each genotype. (**F**) *Zeb1* expression slightly increases upon immobilization. Wild-type and *Zeb1* (+/-) mice were subjected to unilateral hindlimb immobilization during 5 days. At that time, mice were euthanized and *Zeb1* messenger RNA (mRNA) levels were assessed in the immobilized and non-immobilized gastrocnemius by qRT-PCR using *Gapdh* as reference gene. The results are the mean with standard error of at least five mice for each genotype and condition. (**G**) As in (F), but ZEB1 expression was assessed at the protein level by Western blot. Gastrocnemius muscle lysates were blotted for ZEB1 (clone HPA027524) along with GAPDH (clone 14C10) as loading control. See Supplementary Figure S1E for full unedited blots. The blots shown are a representative of three independent experiments. (**H**) As in (F), but the ZEB1 expression was assessed by immunohistochemistry (clone H102) at day 5. Captures are representative of at least five mice for each genotype and condition. Scale bar: 40 μm.

To investigate a potential role of ZEB1 in muscle atrophy, we first examined whether its downregulation has an effect on muscle mass loss in response to immobilization. Two-to-three month-old wild-type and *Zeb1* (+/-) mice were subjected to unilateral hindlimb immobilization for up to 17 days and the weight of both gastrocnemius muscles, from the immobilized and non-immobilized hindlimbs, was assessed over time. As expected, and with respect to the control non-immobilized counterpart, gastrocnemius muscles in the immobilized hindlimb displayed a progressive weight loss (Figure 1A and B). Notably, muscle sparing by immobilization was larger in *Zeb1* (+/-) mice than in wild-type mice (Figure 1A and B). These data indicate that ZEB1 expression protects skeletal muscle from an otherwise excessive atrophy in response to immobilization.

Muscle weight loss during muscle atrophy is accompanied by an increase in the number of smaller size myofibers and a decrease of larger ones (4). Staining with hematoxylin/eosin, and immunofluorescence staining for the structural protein laminin revealed a smaller size in the myofibers of immobilized *Zeb1* (+/-) gastrocnemius muscles compared to wild-type counterparts (Figure 1C and D; Supplementary Figure S1A). Fiber CSA analysis confirmed that upon immobilization *Zeb1* (+/-) muscles contained a larger share of fibers <800 μm^2^ and a lower share of fibers of 800 μm^2^ or more than wild-type muscles (Figure 1E and Supplementary Figure S1B-D).

Next, we examined whether ZEB1 expression is modulated during hindlimb immobilization. Immobilization resulted in a slight increase in ZEB1 messenger RNA (mRNA) and protein (Figure 1F and G; Supplementary Figure S1E). ZEB1 was expressed at the nuclei of some myofibers (Figure 1H and Supplementary Figure S1F) and the number of ZEB1^+^ nuclei in gastrocnemius muscles from both genotypes was similar in the immobilized and non-immobilized hindlimbs (Supplementary Figure S1G).

### ZEB1 inhibits the *in vivo* expression of atrogenes

We next investigated whether ZEB1 regulates the expression of atrogenes. Although muscle weight loss in response to immobilization progressively increases over time, expression of Atrogin-1 and MuRF1 peaks at around day 3 post-immobilization and declines afterward (4).

**Figure 2.**
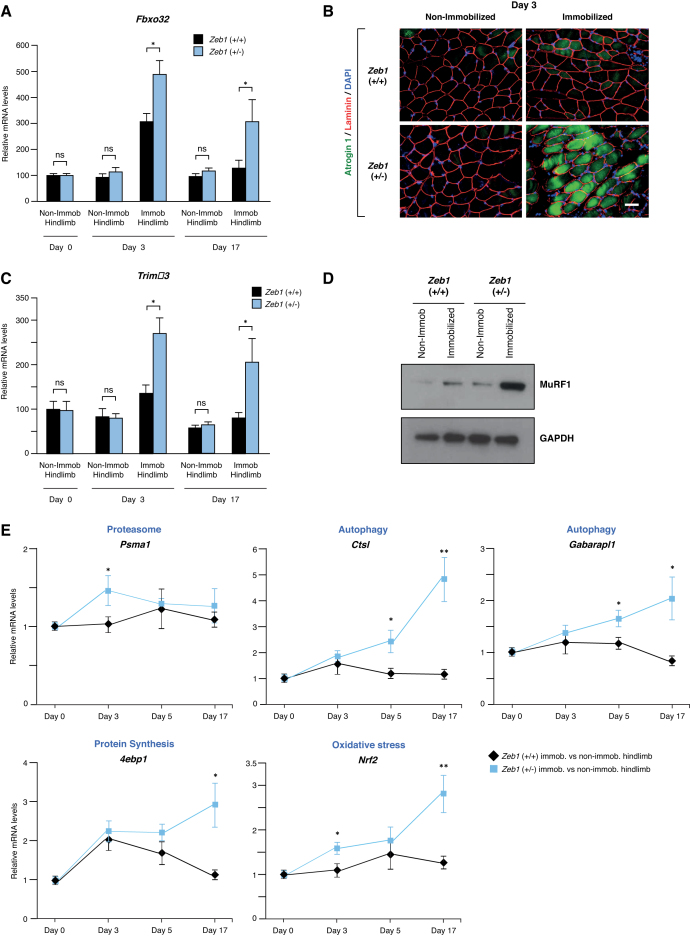
*Zeb1* inhibits the *in vivo* induction of atrogenes upon immobilization. (**A**) Wild-type and *Zeb1* (+/-) mice were subjected to unilateral hindlimb immobilization during 3 and 17 days and their immobilized and non-immobilized gastrocnemius were then examined for *Fbxo32* mRNA expression by qRT-PCR with respect to *Gapdh. Fbxo32* mRNA levels in the non-immobilized hindlimb at day 0 were arbitrarily set to 100 with all other data genotypes and conditions referred to them. Data represent the mean of at least five mice for each genotype and condition. (**B**) The gastrocnemius of mice from both genotypes after 3 days of the unilateral hindlimb immobilization protocol were stained with antibodies against Atrogin-1 (clone AP2041) and laminin (clone 48H-2), and counterstained for 4′,6-diamidino-2-phenylindole (DAPI) for nuclear staining. Captures for single immunostaining are shown in Supplementary Figure S2A. Scale bar: 50 μm. (**C**) As in (A), but for *Trim63*. (**D**) As in (B), but the lysates from gastrocnemius of mice from both genotypes after 3 days of the unilateral hindlimb immobilization protocol were blotted for MuRF1 (clone C11) along with GAPDH (clone 14C10) as loading control. See Supplementary Figure S2B for full unedited blots. The blots shown are representative of three independent experiments. (**E**) Wild-type and *Zeb1* (+/-) mice were subjected to the unilateral hindlimb immobilization protocol for 3, 5 and 17 days. At the end of each time point, they were euthanized and mRNA levels for *Psma1, Ctsl, Gabarapl1, 4ebp1* and *Nrf2* were assessed by qRT-PCR. For each gene, mRNA levels shown correspond to that in the gastrocnemius of the immobilized with respect to the contralateral non-immobilized hindlimb. The gene expression in the non-immobilized gastrocnemius at days 3, 5 and 17 was similar than that at day 0 shown. At least five mice from each genotype and day were analyzed.

Wild-type and *Zeb1* (+/-) mice were subjected to unilateral hindlimb immobilization and their gastrocnemius muscles examined for Atrogin-1/*Fbxo32* mRNA and protein expression. Levels of Atrogin-1/*Fbxo32* expression were similar between the non-immobilized gastrocnemius muscles from both genotypes (Figure 2A and B). However, its induction upon immobilization was larger in *Zeb1* (+/-) muscles (Figure 2A and B; Supplementary Figure S2A). A similar pattern was observed for MuRF1/*Trim63*; non-immobilized gastrocnemius muscles from both genotypes expressed equivalent levels of this atrogene, but immobilization induced higher *Trim63* mRNA and MuRF1 protein levels in *Zeb1* (+/-) gastrocnemius muscles than in wild-type counterparts (Figure 2C and D; Supplementary Figure S2B). Altogether, these results indicate that ZEB1 inhibits Atrogin-1/*Fbxo32* and MuRF1/*Trim63* expression *in vivo*.

Atrogin-1 and MuRF1 are the archetypal atrogenes, but many other genes are induced during muscle atrophy (6–8). The set of atrogenes upregulated in response to different atrophy-inducing conditions is largely, although not completely, overlapping (7,8). We tested whether ZEB1 regulates some of these other atrogenes. The immobilized and non-immobilized gastrocnemius of wild-type and *Zeb1* (+/-) mice were examined for the expression of atrogenes involved in different cellular processes, namely, proteasome system [proteasome subunit, alpha type 1 (*Psma1*)], autophagy [Cathepsin L (*Ctsl*), GABA A-receptor associated protein-like 1 (*Gabarapl1*)], protein synthesis [eukaryotic translation initiation factor 4E binding protein 1 (*4ebp1*)] and oxidative stress [nuclear factor E2 related factor 2 (*Nrf2*)]. Although with different temporal patterns and at lower levels than in the case of *Fbxo32* and *Trim63*, expression of these atrogenes increased in immobilized wild-type gastrocnemius but, as for *Fbxo32* and *Trim63*, their induction was higher in *Zeb1* (+/-) muscles (Figure 2E). Altogether, these results indicate that atrogenes are under negative regulation by ZEB1 whose expression prevents unrestricted atrogene overexpression in response to immobilization.

### ZEB1 inhibits atrogene expression and size reduction in starved C2C12 myotubes

We sought to confirm the role of ZEB1 in muscle atrophy using the C2C12 cell myogenic model, which has been widely employed to study gene expression during both muscle differentiation and atrophy (14,28,29). When grown in high serum (thereafter referred as growth medium), C2C12 cells maintain a proliferating myoblast-like phenotype (see scheme in Supplementary Figure S3A). Only when cells exit the cell cycle upon reaching confluence and are switched into a low-serum medium (differentiation medium) they fuse and form terminally differentiated multinucleated myotubes (28,29). When C2C12 myotubes are starved of serum, glucose and amino acids (atrophic medium), they undergo a rapid reduction in their mean myotube diameter (14).

**Figure 3.**
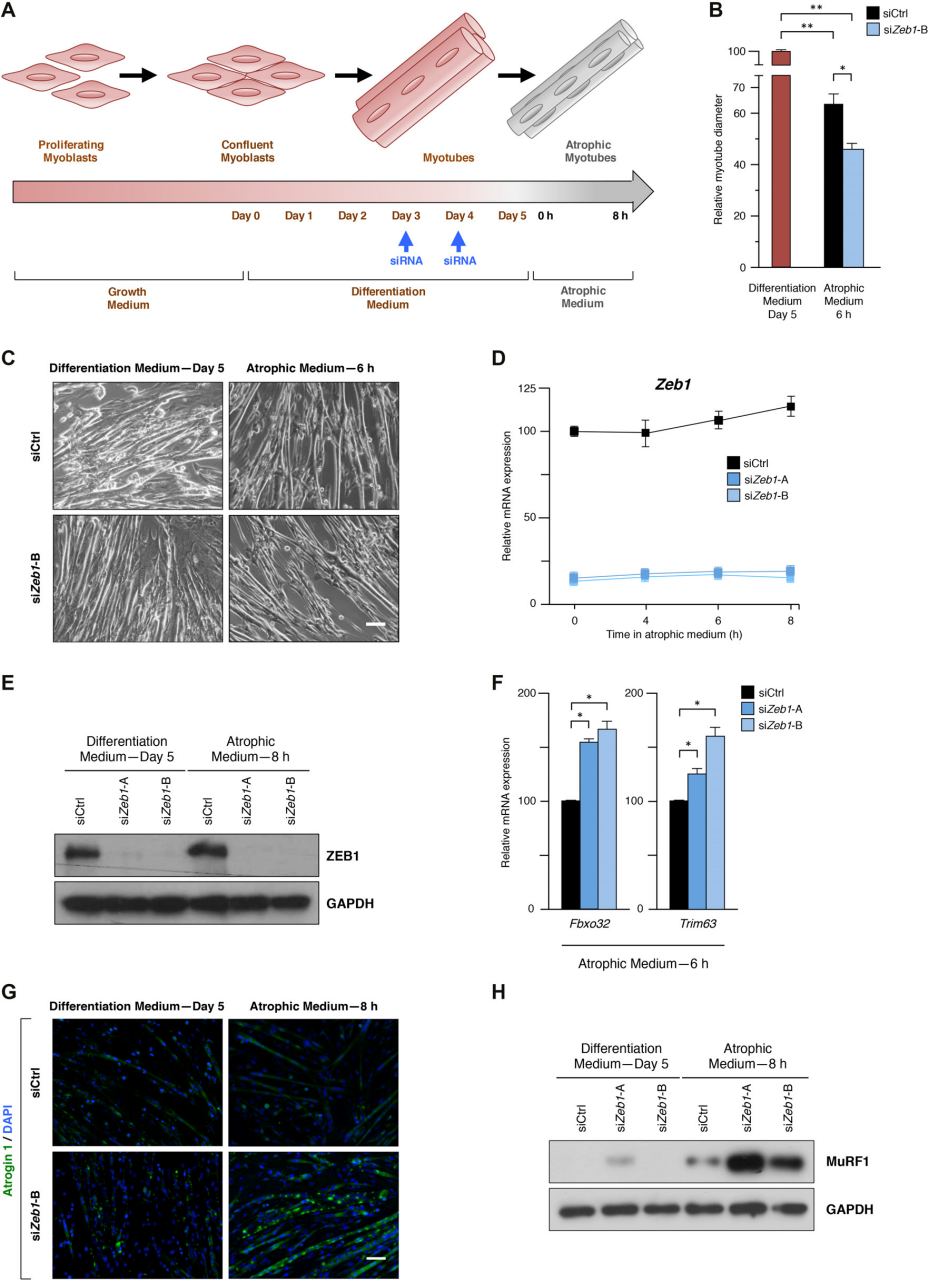
ZEB1 inhibits atrogene expression and size reduction in starved C2C12 myotubes. (**A**) Scheme of the starvation-induced atrophy protocol in C2C12 myotubes. C2C12 myotubes were transfected with siCtrl or any of two siRNA sequences previously validated to specifically knock down *Zeb1* (see Supplementary Figure S3B and C) and their differentiation medium was replaced by atrophic medium for up to 8 h. (**B**) The diameter of C2C12 myotubes subjected to the protocol in (A) was assessed as described in Supplementary Data. Myotube diameter in differentiation medium at day 5 was arbitrarily set at 100. Data represent the average of at least three experiments. (**C**) As in (B), representative captures of C2C12 myotubes transfected with siCtrl or si*Zeb1*-B following incubation in differentiation medium or atrophy medium. Scale bar: 50 μm. (**D**) *Zeb1* mRNA levels in C2C12 myotubes interfered with siCtrl, si*Zeb1*-A or si*Zeb1*-B and cultured in atrophic medium for the indicated periods were assessed by qRT-PCR with respect to *Gapdh. Zeb1* expression in cells interfered with siCtrl at 0 h of atrophic medium was arbitrarily set to 100. Data are representative of four independent experiments. (**E**) As in (C), lysates from C2C12 non-atrophic and atrophic myotubes were assessed by Western blot for ZEB1 expression (clone HPA027524) along with GAPDH (clone 14C10) as loading control. See Supplementary Figure S3C full unedited blots. The blots shown are a representative of four independent experiments. (**F**) As in (A), C2C12 myotubes were interfered with siCtrl, si*Zeb1*-A or si*Zeb1*-B and transferred to atrophy medium. Expression of *Fbxo32* and *Trim63* was assessed by qRT-PCR using *Gapdh* as reference gene. Data represent the average of at least three independent experiments. (**G**) As in (C), but C2C12 non-atrophic and atrophic myotubes were stained for Atrogin-1 (clone AP2041) along with DAPI for nuclear staining. See Supplementary Figure S3D for individual staining. Pictures shown are representative of three independent experiments. Scale bar: 50 μm. (**H**) As in (C), lysates from C2C12 non-atrophic and atrophic myotubes were assessed for MuRF1 expression (clone C11) along with GAPDH (clone 14C10) as loading control. See Supplementary Figure S3E for knockdown of ZEB1 (clone HPA027524) and full unedited blots of the three antibodies. The blots shown are representative of four independent experiments.

At days 3 and 4 of their differentiation, C2C12 myotubes were transfected with either an siRNA control (siCtrl) or any of two siRNA sequences previously validated to specifically knock down *Zeb1* (si*Zeb1*-A, si*Zeb1*-B) (21) (Figure 3A and Supplementary Figure S3B). At day 5, the differentiation medium was replaced by atrophic medium for up to 8 h (Figure 3A). In line with our *in vivo* results above, the diameter reduction induced by the atrophic medium was larger in C2C12 myotubes that had been knocked down for *Zeb1* (Figure 3B and C). Likewise, *Zeb1* mRNA and protein expression slightly increased when C2C12 myotubes were cultured in atrophic medium (Figure 3D and E; Supplementary Figure S3C). Altogether, these data indicate that ZEB1 inhibits muscle atrophy both *in vivo* and in the C2C12 cell model.

The inhibition of atrogenes by ZEB1 was also examined in the C2C12 model. In line with the results above, and compared to C2C12 atrophic myotubes interfered with siCtrl, knockdown of *Zeb1* resulted in higher mRNA and protein levels of Atrogin-1/*Fbxo32* and MuRF1/*Trim63* (Figure 3F–H; Supplementary Figure S3D and E).

### Stage-dependent binding and repression of the *Fbxo32* promoter by ZEB1

Expression of most atrogenes is activated by transcription factors of the *Forkhead box O* (Foxo) family (e.g. FOXO1, FOXO3 and FOXO4) (9,12–14). FOXO3 triggers muscle atrophy through protein degradation via activation of the ubiquitin–proteasome system as well as via autophagy-dependent clearance of organelles (1,2,12–14). The regulatory regions of many atrogenes contain multiple binding sites for FOXO proteins and, accordingly, progressively larger fragments of the *Fbxo32* promoter—that contain an increasing number of FOXO3-binding sites—displayed a parallel larger activation in response to FOXO3 overexpression (Supplementary Figure S4A).

**Figure 4.**
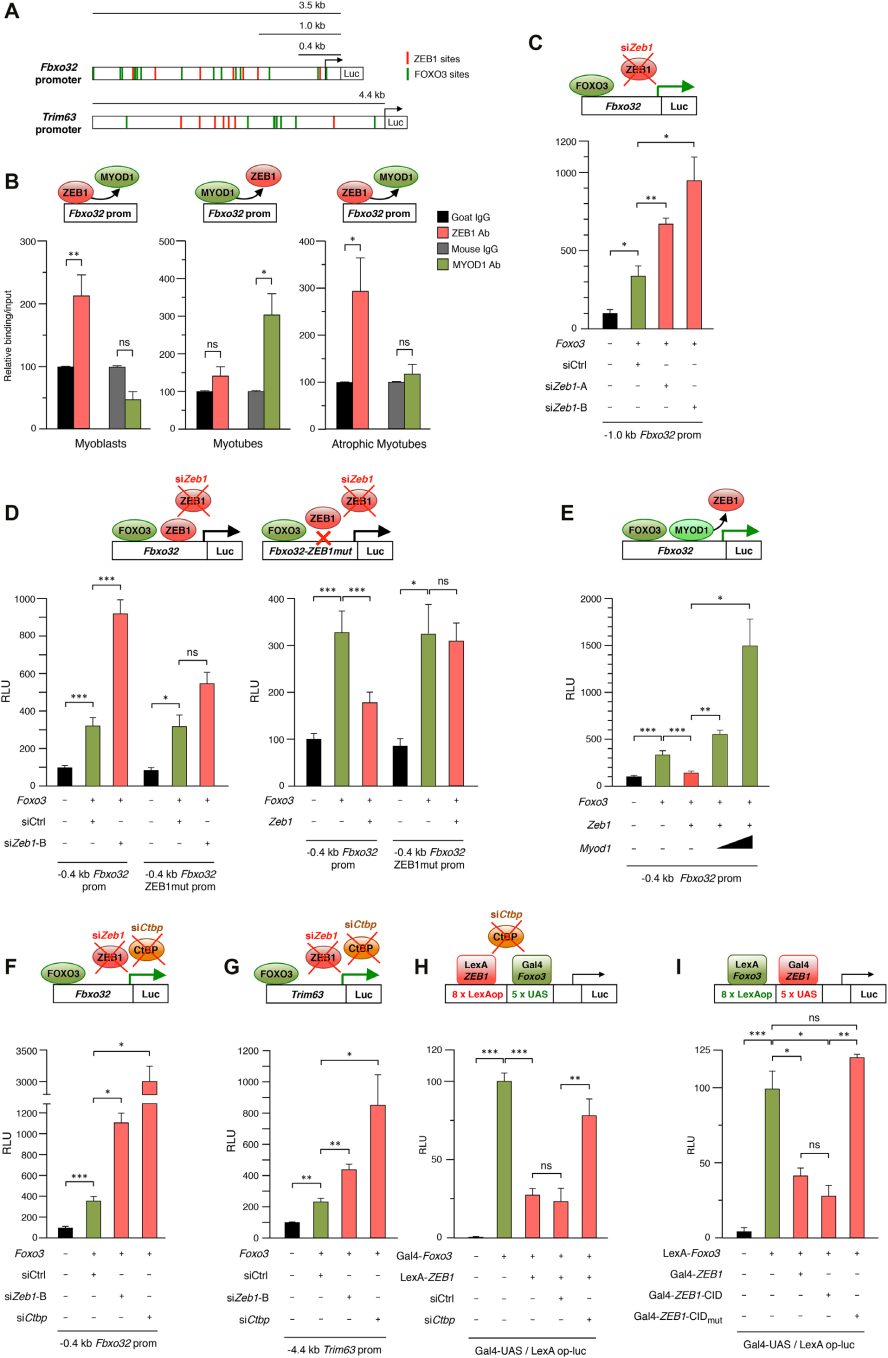
Stage-dependent inhibition of the *Fbxo32* and *Trim63* promoters by ZEB1 is mediated by CtBP-dependent repression of FOXO3 transcriptional activity. (**A**) Schematic representation of the consensus sites for ZEB1 (red boxes) and FOXO3 (green boxes) in the first 3.5 kb and 4.4 kb of the mouse *Fbxo32* and *Trim63* promoters, respectively. Consensus binding sequences for ZEB1 in the *Fbxo32* promoter were identified at –2899 bp, −2584 bp, −1894 bp, −1395 bp, −1254 bp, −1011 bp, and −85 bp. Consensus binding sites for ZEB1 in the Trim63 promoter were identified at −4488 bp, −4444 bp, −3078 bp, −2792 bp, −2566 bp, −2416 bp, −2358 bp, −2254 bp, and –777 bp. Consensus binding sites for FOXO3 in *Fbxo32* and *Trim63* promoters were previously identified in reference (14) or assessed as described in Supplementary Materials and Methods. (**B**) ZEB1 binds to the mouse *Fbxo32* promoter in myoblasts and atrophic myotubes but not in myotubes. DNA from C2C12 myoblasts, myotubes, or atrophic myotubes was immunoprecipitated with antibodies against ZEB1 (clone E-20X), MYOD1 (clone G-1) or their matched IgG controls (goat and mouse IgG, respectively). Immunoprecipitated DNA was then amplified by qRT-PCR in a region of the *Fbxo32* promoter containing a ZEB1 consensus binding site at –85 bp. The condition immunoprecipitated with the IgG control was set to 100. Data represent the average of at least three experiments. (**C**) Transcription of the *Fbxo32* promoter is under negative regulation by endogenous ZEB1. 0.48 μg of a luciferase reporter containing a 1.0 kb fragment of the mouse *Fbxo32* promoter (14) was co-transfected in C2C12 cells along with 0.82 μg of an expression vector for FOXO3 (or the corresponding molar amount of the empty expression vector) to induce *Fbxo32* transcription. Throughout this Figure, the effect of overexpressing the indicated genes (*Foxo3* in this panel) is shown with respect to their corresponding empty vectors. Where indicated, cells were also transfected with 50 nM of either siCtrl, si*Zeb1*-A or si*Zeb1*-B. Transfections and assessment of Relative luciferase units (RLU) were performed as described in Supplementary Information. The first condition was arbitrarily set to 100. Data represent the average of three independent experiments. (**D**) *Left panel:* As in (C) but cells were instead transfected with 0.43 μg of either a luciferase reporter containing a 0.4 kb fragment of the mouse *Fbxo32* promoter (14) or version of it where only the ZEB1 binding site at -85 bp has been mutated to a sequence known to not bind ZEB1 (see Supplementary Information for details). *Right panel:* As in the left panel, but 1.88 μg of an expression vector for *Zeb1* (or the corresponding molar amount of the empty expression vector) were also transfected. The first condition was arbitrarily set to 100. Data represent the average of three independent experiments. (**E**) Overexpression of MYOD1 displaces ZEB1 from its binding to the 0.4 kb *Fbxo32* luciferase reporter. As in the right panel of (D) but 0.04 μg or 0.13 μg of an expression vector for *Myod1* (or the corresponding molar amount of the empty expression vector) were transfected along with *Zeb1*. The first condition was arbitrarily set to 100. Data shown are the mean of three independent experiments. (**F**) Transcription of the *Fbxo32* promoter is under negative regulation by endogenous CtBP. As in (D) but cells were transfected with a siRNA against *Ctbp*. The first condition was arbitrarily set to 100. Data represent the average of three independent experiments. (**G**) As in (F) but cells were instead transfected with 0.77 μg of a luciferase reporter containing a 4.4 kb fragment of the mouse *Trim63* promoter. The first condition was arbitrarily set to 100. Data are the average of at least three independent experiments. (**H**) FOXO3 transcriptional activity is repressed by ZEB1 and CtBP. 293T cells were transfected with 0.50 μg of a reporter containing LexA operon and Gal-UAS sites (L8G5-luc) along with 1.04 μg Gal4-*Foxo3* and/or 1.15 μg LexA-*ZEB1* (or their corresponding empty vectors). Where indicated, cells were transfected with 10–20 nM of either siCtrl or si*Ctbp*. The condition overexpressing only Gal4-*Foxo3* was arbitrarily set to 100. Data represent the average of five independent experiments. (**I**) ZEB1 represses FOXO3 transcriptional activity through a CtBP-dependent mechanism. As in (H) but the Gal4 and LexA fusion proteins were swapped: *ZEB1, ZEB1*-CID and *ZEB1*-CID_mut_ were fused to Gal4 whereas *Foxo3* was fused to LexA. Cells were transfected with 0.50 μg of L8G5-luc, 0.70 μg of LexA-*Foxo3*, 1.50 μg Gal4-*ZEB1*, 0.79 μg of Gal4-*ZEB1*-CID and/or *ZEB1*-CID_mut_. The condition overexpressing only LexA-*Foxo3* was arbitrarily set to 100. Data are the average of three independent experiments.

ZEB1 regulates gene expression by binding to E-box and E-box-like sequences (CANNTG) in the regulatory regions of its target genes (30,31). Analysis of the *Fbxo32* and *Trim63* promoters revealed the existence of several consensus binding sites for ZEB1, particularly in the former where many FOXO3 consensus sites are located in close proximity to ZEB1’s (Figure 4A). ZEB1 and MYOD1 partially overlap in their DNA sequence recognition (30–32), with ZEB1 repressing key muscle differentiation genes in a reverse temporal pattern *vis-à-vis* MYOD1 (20,21). Thus, during the myoblast stage, ZEB1 binds to E-boxes in the promoters of differentiation genes and represses their transcription, but, as differentiation proceeds, MYOD1 accumulates and displaces ZEB1 from these E-boxes (20,21).

To investigate whether ZEB1 regulation of *Fbxo32* involves direct binding to its promoter, we examined ZEB1’s capacity to bind to a consensus binding site located at −85 bp of the *Fbxo32* promoter in myoblasts, myotubes and atrophic myotubes. Interestingly, we found that in myoblasts and atrophic myotubes, but not in non-atrophic myotubes, an anti-ZEB1 antibody—but not its specie-matched IgG control—immunoprecipitated a fragment of the *Fbxo32* promoter containing the −85 bp binding site (Figure 4B). This stage-specific binding of ZEB1 to the *Fbxo32* promoter was reversely mirrored by the pattern of binding of MYOD1; an anti-MYOD1 antibody—but not its respective IgG control—immunoprecipitated the *Fbxo32* promoter in myotubes, but not in atrophic myotubes or in myoblasts.

We next examined the transcriptional activity of the *Fbxo32* promoter following either the knockdown or overexpression of *Zeb1*. C2C12 cells were transfected with 0.4 and 1.0 kb fragments of *Fbxo32* promoter fused to luciferase along with an expression vector for FOXO3 to induce its transcription. As expected, FOXO3 activated both *Fbxo32* promoter reporters (Figure 4C and D). Compared to siCtrl, si*Zeb1*-A and si*Zeb1*-B further increased FOXO3-mediated induction of the *Fbxo32* promoter (Figure 4C and left panel of Figure 4D), indicating that the *Fbxo32* promoter is under negative transcriptional regulation by endogenous ZEB1. In turn, exogenous overexpression of *Zeb1* downregulated FOXO3-mediated induction of the *Fbxo32* promoter luciferase reporter (Figure 4D, right panel). When *Foxo3* was knocked down with a specific siRNA, overexpression of *Zeb1* had no significant effect on the transcriptional activity of the 0.4 kb *Fbxo32* promoter reporter (Supplementary Figure S4B and C). Mutation of the ZEB1-binding site at the −85 bp site in the context of the 0.4 kb *Fbxo32* luciferase reporter to a sequence known not to bind ZEB1 reduced the effect of both *Zeb1* knockdown and *Zeb1* overexpression on *Fbxo32* transcription (Figure 4D). ZEB1-mediated repression of the 0.4 kb *Fbxo32* promoter reporter was also reverted by overexpression of MYOD1 (Figure 4E).

### ZEB1 inhibits *Fbxo32* and *Trim63* promoters through CtBP-dependent repression of FOXO3 transcriptional activity

ZEB1 represses transcription of its target genes by recruitment of non-DNA binding transcriptional co-repressors, chiefly of CtBP (21,25,27,33, and reviewed in 23). In that line, an siRNA against *Ctbp* increased *Fbxo32* promoter activity (Figure 4F). The large increase in *Fbxo32* transcription induced by si*Ctbp* suggests that *Fbxo32* is under negative regulation by other CtBP-binding factors besides ZEB1. ZEB1 repression of *Fbxo32* was also partially relieved by blocking of CtBP activity with 2-keto-4-methylthiobutyrate (MTOB), an intermediate in the methionine salvage pathway that binds and inactivates CtBP (33,34) (Supplementary Figure S4D).

Next, we examined the potential regulation of *Trim63* by ZEB1 at the transcriptional level. Knockdown of *Zeb1* and *Ctbp* upregulated FOXO3-induced transcription of the *Trim63* reporter (Figure 4G), indicating that, as for *Fbxo32*, MuRF1 expression is inhibited at the transcriptional level by endogenous ZEB1 and CtBP.

ZEB1 repression of several atrogenes (Figure 2) suggests that ZEB1 modulates the activity of a common activator of muscle atrophy. The results above also indicate that ZEB1 represses FOXO3-induced activation of the *Fbxo32* and *Trim63* promoters. We therefore investigated whether ZEB1 directly represses FOXO3-mediated transcriptional activity using a heterologous luciferase reporter (L8G5-luc) that contains binding sites for yeast Gal4 (Gal4-UAS) and bacterial LexA (LexAOp) proteins (scheme on top of Figure 4H). The cDNA of *Foxo3* fused to the DNA-binding domain of Gal4 (Gal4-*Foxo3*) activated the basal transcription of the L8G5-luc reporter (Figure 4H). In turn, the cDNA of *ZEB1* fused to the DNA-binding domain of LexA (LexA-*ZEB1*) repressed Gal4-*Foxo3*-induced transcriptional activation of the L8G5-luc reporter (Figure 4H). In line with the results above with the *Fbxo32* and *Trim63* promoters (Figure 4F and G), knockdown of *Ctbp* with an siRNA partially relieved the repression of Gal4-*Foxo3* by LexA-*ZEB1* (Figure 4H). A similar result was obtained when the cDNA of *Foxo3* was instead fused to the DNA-binding domain of LexA and that of ZEB1 to Gal4 (Figure 4I). *Foxo3*-mediated transcription in this heterologous system was also repressed by a ZEB1 fragment containing only its CtBP-interacting domain (CID) fused to Gal4 (Gal4-*ZEB1*-CID) (Figure 4I). However, mutation of the three CtBP sites within ZEB1’s CID (Gal4-*ZEB1*-CID_mut_) abrogated transcriptional repression of FOXO3 by *ZEB1*-CID. The conclusions from these results are twofold: first, ZEB1 inhibits *Foxo3-*mediated induction of atrogenes; and second, ZEB1 has the intrinsic capacity to repress *Foxo3* transcriptional activity through, at least in part, the recruitment of the CtBP co-repressor.

### *In vivo* repression of the *Fbxo32* promoter by endogenous ZEB1

**Figure 5.**
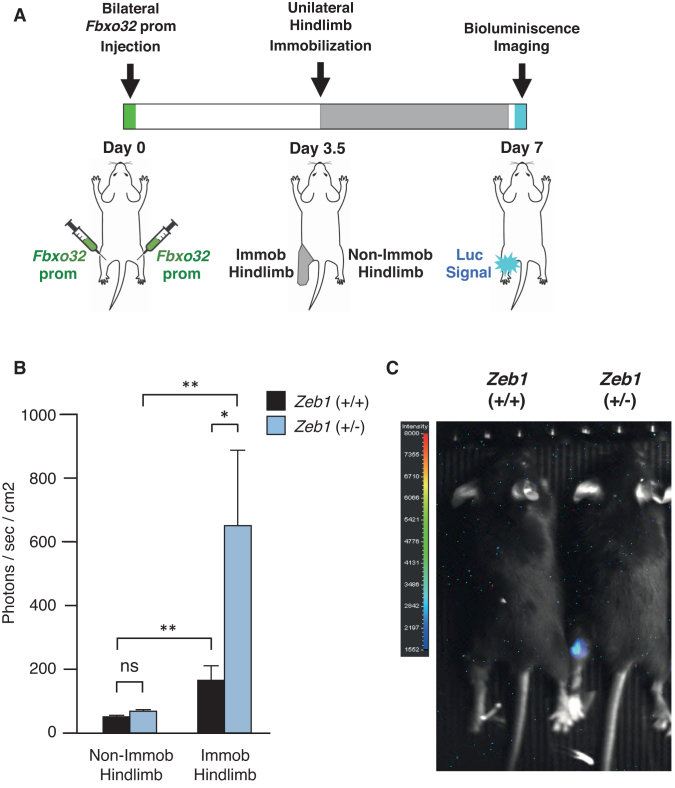
*In vivo* repression of the *Fbxo32* promoter by endogenous ZEB1. (**A**) Graphic representation of the protocol for the *in vivo* assessment of ZEB1 regulation of the *Fbxo32* promoter. Both hindlimbs of wild-type and *Zeb1* (+/-) mice were injected with a 3.5 kb fragment of the *Fbxo32* promoter fused to luciferase (14). After 3.5 days, mice were subjected to unilateral (left) hindlimb immobilization for 3.5 additional days. At day 7, *Fbxo32* promoter activity was assessed *in vivo* by whole-body bioluminescence imaging. See Supplementary Data for details. (**B**) ZEB1 inhibits the *Fbxo32* promoter *in vivo*. In both genotypes, the bioluminescence signal emitted by the *Fbxo32* promoter is higher in the immobilized hindlimb than in the non-immobilized hindlimb. However, immobilization induced greater bioluminescence signal in *Zeb1* (+/−) mice than in wild-type mice. Data represent the average of seven mice of each genotype. (**C**) Bioluminescence signal rendered by a representative mouse for each genotype at day 7.

Lastly, we examined the *in vivo* regulation of the *Fbxo32* promoter by endogenous ZEB1 (see scheme in Figure 5A). Both hindlimbs of wild-type and *Zeb1* (+/-) mice were injected with the *Fbxo32* promoter fused to luciferase. After 3.5 days, the left hindlimb was immobilized during 3.5 additional days, while the right hindlimb remained non-immobilized. At day 7, luciferase signal emission was assessed by whole-body bioluminescence imaging. In line with the above results, the luminescence signal emitted by the *Fbxo32* promoter was higher in the immobilized hindlimb of *Zeb1* (+/-) mice than in that of wild-type counterparts. These results indicate that endogenous ZEB1 also inhibits the transcription of the *Fbxo32* promoter *in vivo* (Figure 5B and C).

## DISCUSSION

**Figure 6.**
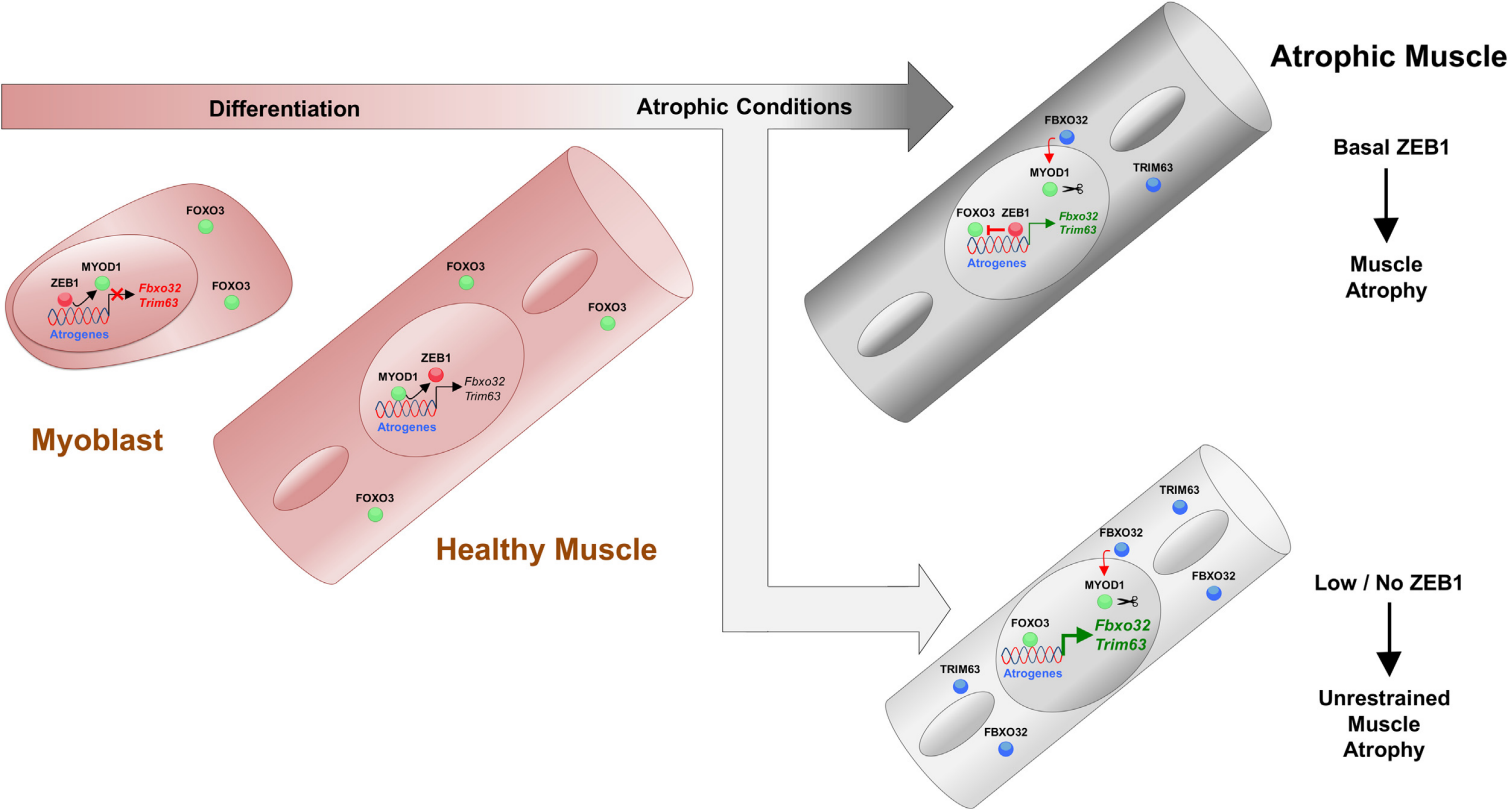
Summary model: ZEB1 inhibits muscle atrophy and atrogene expression in a stage-dependent manner through CtBP-mediated repression of FOXO3 transcriptional activity. See main text for details.

The transcriptional regulation of muscle atrophy is still not completely understood. This study showed that ZEB1 inhibits muscle atrophy and atrogene expression (see summary model in Figure 6). Full levels of ZEB1 expression protected skeletal muscle from an otherwise unrestrained muscle atrophy and atrogene overexpression in response to immobilization as occurs when ZEB1 levels are reduced. In the C2C12 myogenic model, ZEB1 knockdown upregulated Atrogin-1 and MuRF1 expression and enhanced the reduction in myotube diameter triggered by growth factor starvation. At the mechanistic level, ZEB1 directly binds to the *Fbxo32* promoter in a stage-dependent manner and represses its transcription and that of *Trim63*—both in cell systems and/or *in vivo*—through CtBP-dependent inhibition of FOXO3 transcriptional activity.

The molecular mechanisms controlling fiber size under homeostasis and during atrophy are different (3), and we found here that under basal (non-immobilized) conditions, *Zeb1* (+/-) muscles display equivalent weight and expressed similarly low or non-existing levels of Atrogin-1 and MuRF1 as wild-type counterparts. This can be explained because in homeostatic (non-immobilized) conditions, ZEB1 does not bind to the *Fbxo32* promoter that it is instead occupied by MYOD1. Nevertheless, binding of MYOD1 does not seem to be sufficient to induce *Fbxo32* expression; transcriptional activation of atrogenes requires of FOXO3, which remains translocated to the cytoplasm in non-immobilized muscles (2,3,14).

In turn, a partial downregulation of *Zeb1*—to around half the levels with respect to that in wild-type mice—was sufficient to trigger enhanced muscle atrophy in response to immobilization in *Zeb1* (+/-) muscles. In addition, immobilization induced a moderate increase in ZEB1 mRNA and protein expression. This would suggest that the protecting role of ZEB1 against unrestrained muscle atrophy during immobilization depends on a fine threshold of its expression. Interestingly, an analogous expression threshold has been reported for ZEB1 tumor-promoting functions. Thus, a partial downregulation of *Zeb1* in either cancer cells or stromal cells of the tumor microenvironment is enough to completely block the malignant progression of lung, colon and ovarian carcinomas in *Zeb1* (+/-) mice (33,35,36). In addition, ZEB1 transcriptional activity is regulated by *cis* and *trans* mechanisms that determine its binding to target gene promoters and its recruitment of transcriptional co-activators and co-repressors (15). Muscles need to continuously and finely regulate their protein synthesis and proteolysis in response to atrophic and hypertrophic signals. In that regard, a multifunctional and tightly regulated protein like ZEB1 can play such role as a stage-dependent and discerning modulator of muscle loss.

ZEB1 inhibited muscle atrophy in a stage-dependent manner; ZEB1 bound to the *Fbxo32* promoter in atrophic myotubes, but not in non-atrophic myotubes, thus contributing to explain the lack of atrophy and atrogene upregulation in *Zeb1* (+/-) muscles under basal (non-immobilized) conditions. Regulation of muscle differentiation by ZEB1 and other EMT factors (e.g. SNAI1/2) also occurs in a stage-dependent manner (20,21,37). ZEB1 and SNAI1/2 share DNA-binding sites (E-box and E-box-like sequences) with MYOD1 in the promoters of muscle differentiation genes. During the myoblast stage, ZEB1 and SNAI1/2 occupy these promoters to repress their expression, but as muscle differentiation proceeds, MYOD1 accumulates and displaces EMT factors from these genes activating their expression (20,21,37). We found here a similar reverse binding pattern of ZEB1 and MYOD1 with respect to atrogenes. ZEB1 was excluded from the *Fbxo32* promoter in non-atrophic myotubes where MYOD1 was instead occupying the promoter. MYOD1 has higher affinity than ZEB1 for binding to E-boxes (20) and, accordingly, overexpression of MYOD1 was able to displace ZEB1 from the *Fbxo32* promoter. In that regard, the preferred binding of ZEB1 over MYOD1 to the *Fbxo32* promoter in atrophic myotubes is probably related not only to the slight upregulation of ZEB1 in atrophic muscles and myotubes (Figures 1F, G, and 3D), but also to the downregulation of *Myod1* mRNA during atrophy (Supplementary Figure S4E) and the reported role of Atrogin-1 targeting MYOD1 protein for ubiquitin degradation (38,39) (see model in Figure 6).

ZEB1 repressed the *Fbxo32* promoter through a mechanism that involved recruitment of CtBP and inhibition of FOXO3 transcriptional activity. Despite that among all transcription factors CtBP has one of the highest affinity for ZEB1 (40), *CtBP* knockdown upregulated *Fbxo32* and *Trim63* promoters transcription above the effect of *Zeb1* knockdown, suggesting that Atrogin-1 and MuRF1 expression are under negative regulation by CtBP-binding transcription factor(s) other than ZEB1.

Notably, muscle atrophy-inducing conditions of very disparate origins—from immobilization or denervation to cancer cachexia, fasting or uremia—upregulate a highly overlapping set of atrogenes (7–9). It remains to be elucidated whether ZEB1 represses all atrogenes or only a subset. Nevertheless, data shown here indicate that, in addition to the E3 ubiquitin ligases *Fbxo32* and *Trim63*, ZEB1 also represses other components of the ubiquitin–proteasome chain (*Psma1*), members of the autophagy-lysosomal system (*Ctsl, Gabarapl1*), as well as genes involved in protein synthesis (*4ebp1*), and oxidative stress (*Nrf2*). Although FOXO3 is required for muscle atrophy and a majority of atrogenes are induced by FOXO proteins, their dependence on FOXO is determined by the atrophy-inducing condition; thus, *Nrf2* is induced by FOXO proteins upon muscle denervation, but not in response to fasting (9). This draws a nuance model of transcriptional regulation of atrogenes where other transcriptional activators, beyond FOXO proteins, may also induce atrogene expression. It is also possible that ZEB1 represses atrogenes that are independent of FOXO3. ZEB1 represses the activity of a wide range of transcriptional activators with its inhibitory effect and the mechanism involved determined by the promoter, the co-repressors it recruits and the activation/differentiation stage of cells (23,24,26,27,41). In addition, ZEB1 can also function as a transcriptional activator; binding of ZEB1 to the histone acetyltransferase p300 acetylates the CID region of ZEB1, thus displacing CtBP (27,42). In that line, in B lymphocytes, ZEB1 synergizes with FOXO3, rather than repressing it, in the activation of cell cycle genes cyclin G2 (*Ccng2)* and p130 (*Rbl2*) (43), highlighting once again the promoter and cell-type specificity of the link between ZEB1 and FOXO3.

This study unveiled an unexpected role for ZEB1 beyond cell differentiation and cancer. The identification here of ZEB1 as an inhibitor of atrogene expression offers new approaches for therapies aimed at preventing or treating muscle atrophy.

## Supplementary Material

Supplementary Data
