# Supplementary material for: Regulation of muscle atrophy-related genes by the opposing transcriptional activities of ZEB1/CtBP and FOXO3
Source: Nucleic Acids Res. 2018 Oct 10;46(20):10697–708. doi: 10.1093/nar/gky835 (PMC6237734; doi:10.1093/nar/gky835)
Supplement: Supplementary Data [file gky835_supplemental_files.pdf]

SUPPLEMENTARY DATA

Regulation of muscle atrophy-related genes by the opposing transcriptional activities of ZEB1/CtBP and FOXO3

Chiara Ninfali [1], Laura Siles [1], Douglas S. Darling [2], Antonio Postigo [1,3,4,*]

[1] Group of Transcriptional Regulation of Gene Expression, IDIBAPS, Barcelona 08036, Spain
[2] Center for Genetics and Molecular Medicine and Dept. of Immunology and Infectious Diseases, University of Louisville, KY 40202, USA
[3] Molecular Targets Program, James G. Brown Cancer Center, Louisville, KY 40202, USA
[4] ICREA, Barcelona 08010, Spain

* **Address correspondence to**: A Postigo, Group of Transcriptional Regulation of Gene Expression. IDIBAPS. Casanova 143. 08036 Barcelona, Spain. Email: idib412@clinic.cat

SUPPLEMENTARY MATERIALS AND METHODS

Mouse models and hindlimb immobilization protocol

The following models were used: C57BL/6 [referred throughout as either wild-type or *Zeb1* (+/+)] (Jackson Laboratories) and *Zeb1* (+/-) (24). Unilateral hindlimb immobilization was performed by covering one of the hindlimbs with a plastic cast for different periods as described elsewhere (11). At the end of the protocol, mice were sacrificed and the gastrocnemius muscle extracted for further analyses.

Cell lines, cell culture, and C2C12 myotube differentiation and starvation

C2C12 and 293T cells were obtained from the American Type Culture Collection (ATCC)-LGC Standards (Middlesex, England, UK). C2C12 cells were plated on 12-well plates and cultured in Dulbecco's modified Eagle Medium (DMEM) (Lonza, Basel, Switzerland) supplemented with 15% Fetal Bovine Serum (Sigma-Aldrich. St. Louis, MO), and 1% penicillin-streptomycin (Pen/Strep) (Lonza). This media is referred hereafter as Growth Medium. When cells reached confluence, the medium was replaced with DMEM supplemented with 2% of Horse serum (Sigma-Aldrich) and 1% Pen/Strep (referred hereafter as Differentiation Medium) for an additional 4 days. For starvation, C2C12 myotubes were placed in DMEM without glucose (Lonza) (referred as Atrophic Medium) for different periods. 293T cells were grown in Growth Medium but containing only 10% FBS. For knockdown of gene expression, C2C12 and 293T cells were plated onto 12-well plates and transfected with a control siRNA (siCtrl) or specific siRNA oligonucleotides against the gene of interest (Supplementary Table S1) using 4 μl of RNAiMAX (Thermo-Fisher Scientific, Carlsbad, CA). After 48 h, cells were collected and processed for further analysis.

Plasmids

Firefly luciferase reporters used in the study were obtained from the following researchers: wild-type 0.4, 1.0, and 3.5 kb fragments of the mouse *Fbxo32* promoter fused to firefly luciferase (pGL3pAT1-0.4, pGL3pAT1-1.0, and pGL3pAT1-3.5, respectively) were obtained from SH Lecker (Harvard Medical School, Boston, MA, USA) (19); wild-type 4.4 kb fragment of mouse *Trim63* promoter subcloned into the pGL3 firefly luciferase vector from B-C Oh (Lee Gil Ya Cancer and Diabetes Institute, Incheon, Korea) (4); and, a heterologous luciferase reporter (L8G5-luc) containing five binding sites for yeast Gal4 (UAS) and eight sites for bacterial LexA (LexAOp) proteins from J-L Baert (Université des Sciences et Technologies, Lille, France) (10,12). In addition, the following expression vectors were used: pECE-*Foxo3* from P Coffer (University Medical Center Utrecht, The Netherlands) (25), pEMSV-MyoD from AB Lassar (Dana-Farber Harvard Cancer Center, Harvard University, Boston, MA, USA) (6), pcDNA3-*Zeb1* from K Miyazono (University of Tokyo, Japan) (21), Gal4 and Gal4-*Foxo3* from H Ito (Keio University School of Medicine, Tokyo, Japan) (14); LexA (PBXL3), LexA-*ZEB1* (PBXL3-*ZEB1*), LexA-*ZEB1*-CID (PBXL3-*ZEB1*-CID), and LexA-*ZEB1*-CID_{mut} (PBXL3-*ZEB1*-CID_{mut}), (16,17), and LexA-Foxo3 from FM Stanley (New York University School of Medicine, NY, USA) (9). SV40-β-galactosidase and pBluescript SK vectors were obtained commercially from Clontech (Takara, Kyoto, Japan), and Stratagene (Agilent, Santa Clara, CA), respectively.

Immunohistochemistry

Gastrocnemius muscles were dissected, mounted on corks, embedded in Tissue-Tek® O.C.T. Compound (Electron Microscopy Sciences, Hatfield, PA, USA), and frozen in liquid nitrogen cooled isopentane and stored at -80°C. Next, 8 μm cryosections were prepared (Leica Cryostat CM 1950, Leica Biosystems, Wetzlar, Germany), fixed for 20 min in ice-cooled acetone and permeabilized in 0.25% Triton X-100 in PBS for 30 min. At least one slide from each sample used for immunohistochemistry (IHC) was routinely stained with hematoxylin and eosin. To block endogenous peroxidase, slides of mouse muscle samples were incubated with 0.3% hydrogen peroxide in PBS and with a non-specific binding blocking solution (NSBBS) [5% goat normal serum (Jackson ImmunoResearch Europe Ltd., Cambridgeshire, England, UK), 4% BSA in PBS, 0.5% Tween 20] followed by the corresponding primary (overnight at 4°C) and HRP-conjugated secondary (1 h at room temperature) antibodies. ZEB1 antibody (clone H102) was used at 1/100 dilution. The immunohistochemistry reaction was developed with a DAB substrate kit (Vector Labs, Burlingame, CA) before slides were counterstained with hematoxylin and mounted in Di-N-butylPhthalate in Xylene solution (DPX,

Sigma-Aldrich). Staining was evaluated in a Nikon Olympus BX41 microscope and images processed with ImageJ software (NIH, Bethesda, MD).

Immunofluorescence

Gastrocnemius muscles from mice were embedded, frozen, cryo-sectioned and fixed as described for IHC. Fixed samples of gastrocnemius muscles, were then incubated with 0.1% $NaBH_4$ to block non-specific autofluorescence and with NSBBS to reduce non-specific antibody signal. Slides were then incubated with the corresponding primary (overnight at 4°C) and fluorochrome-conjugated secondary (1 h at room temperature) antibodies. The primary antibodies listed in Supplementary Table S2 were used as follows: Atrogin-1 (AP2041, 1/100 dilution), and laminin (48H-2, 1/80). Their respective secondary antibodies were used as follows: anti-rabbit Dylight 488 (1/250), and donkey anti-rat rodamine Red-X (1/250). Slides were then mounted with Prolong Gold® Antifade Reagent with DAPI (Thermo Fisher). Staining was evaluated in a Nikon Eclipse E600 microscope (Minato, Tokyo, Japan). For immunofluorescence experiments in C2C12 myotubes, cells were plated onto 12-well plates, washed with PBS and permeabilized in 0.25% Triton X-100 in PBS for 30 min. After washing cells again with PBS, myotubes were first incubated for 1-3 h with the same blocking NSBBS solution described above followed by overnight incubation at 4°C with a polyclonal antibody against Atrogin-1 (AP2041, dilution 1/100). Wells were then washed with PBS, incubated for 90 min with a donkey anti-rabbit Dylight 488 (1/250 in blocking solution), washed again and a drop of Prolong Gold® Antifade Reagent with DAPI (Thermo Fisher) was added before visualization in an inverted Zeiss Axiovert 200 (Oberkochen, Germany) microscope. All immunofluorescence images were processed with ImageJ software (NIH, Bethesda, MD).

Assessment of myofiber cross-sectional area and C2C12 myotube diameter

Gastrocnemius samples from wild-type and *Zeb1* (+/-) after 5 and 17 days of unilateral hindlimb immobilization were assessed for myofiber area (referred as cross-sectional area) as described elsewhere (13). Briefly, samples were stained for hematoxylin/eosin and the area of at least 160 fibers was measured using ImageJ software (NIH, Bethesda, MD). The diameter of C2C12 myotubes cultured in either differentiation or atrophic medium was quantified using ImageJ software. The diameter of each individual myotube was calculated as the average of ten measurements along its length. At least 100 myotubes from ten fields at 20X magnification were assessed.

RNA extraction and qRT-PCR analysis

Total RNA from gastrocnemius muscles and C2C12 cells was extracted using TRIzol (Life Technologies, Thermo Fisher). RNA was retrotranscribed with random hexamers using High-Capacity cDNA Reverse Transcription kit (Applied Biosystems, Thermo Fisher). mRNA levels were determined by qRT-PCR using SYBRGreen GoTaq® qPCR Master Mix (Promega Corp., Madison, WI) in a LightCycler ® 96 real-time PCR apparatus (Roche, Rotkreuz, Switzerland). Results were analyzed using LightCycler 96 SW1.1 software (Roche) by the ΔΔCt method using *Gapdh* as reference gene. Primers used to amplify the different genes examined in the study are detailed in Supplementary Table S3.

Western blots

C2C12 myotubes and surgically dissected gastrocnemius were washed with ice-cold PBS and resuspended in RIPA lysis buffer (150 mM NaCl, 50 mM Tris pH 8.0 0.1% NP40, 0.5 % SDS, 2 mM EDTA) containing protease inhibitors (10 µg/ml aprotinin, leupeptin, pepstatin A and PMSF) as previously described (22). Lysates were sonicated in a Sonics Vibra-Cell™ CV188 instrument (Misonix Inc., Farmingdale, NY), clarified by centrifugation and quantified by Bradford assay. Lysates were then boiled and loaded onto 10 % polyacrylamide gels and transferred to a PVDF membrane (Immobilon-P, Millipore, Bedford, MA, USA). Membranes were blocked with 5 % non-fat milk in TBST (20 mM Tris pH 7.5, 150 mM NaCl, 0.1% Tween 20) buffer and split into two sections at the level indicated in the Figure Legend. The upper section was used to detect ZEB1 (HPA027524, dilution 1/500) while the lower section was used to detect MuRF1 (C-11, 1/900) and GAPDH (14C10, 1/4000). After washing several times with TBST buffer, membranes were incubated with their respective HRP-conjugated secondary antibodies. The chemiluminescence reaction was developed with Clarity Western ECL (Bio-Rad). Full unedited images for all Western blots

in the study are included as Supplementary Figures with a box indicating the area displayed in the main Figure.

Promoter analysis and sited directed mutagenesis of the *Fbxo32* promoter

Consensus binding sites for ZEB1 in mouse *Fbxo32* and *Trim63* promoters were identified using MacVector 16.0.8 software (MacVector Inc, Apex, NC). FOXO3 binding sites in both promoters were described elsewhere (19) and/or identified using MacVector 16.0.8 software. The ZEB1 binding site at -85 bp (CACGTG) of the 0.4 kb *Fbxo32* promoter luciferase reporter (19) was mutated to a sequence (CACTCA) known not to bind to ZEB1 (20). Site-directed mutagenesis was performed as previously described (18). Briefly, the 0.4 kb *Fbxo32* luciferase reporter was amplified using *PfuUltra* High-Fidelity DNA polymerase (Stratagene-Agilent) and oligos described in Supplementary Table S4. DNA was then digested with DpnI and transformed into chemically competent bacteria.

Chromatin Immunoprecipitation Assays

Chromatin immunoprecipitation (ChIP) assays were performed using EpiQuick ChIP kit (Epigentek) as per manufacturer's instructions. Briefly, C2C12 myoblasts, starved, and non-starved myotubes were incubated for 20 min with 1% formaldehyde solution (Electron Microscopy) at room temperature followed by incubation with 1.25 mM glycine. Lysates were sonicated as described elsewhere (18). Goat anti-mouse/human ZEB1 (4 μg, clone E-20X), mouse MYOD1 (4 μg, clone G-1) and their corresponding normal goat and mouse IgG (4 μg, Jackson ImmunoResearch Europe Ltd.), respectively, were used. Design of primers for qRT-PCR was conducted using MacVector software (MacVector Inc, Apex, NC). DNA fragments were quantified by qRT-PCR as detailed above using the primers detailed in Supplementary Table S5. In all qRT-PCRs, values shown represent relative binding in relation to input and are the average of at least three independent ChIP experiments, each one performed in triplicate.

Cell line-based transcriptional assays

C2C12 and 293T cells were seeded in 12-well plates and transiently transfected with firefly luciferase reporters for *Fbxo32*, *Trim63* or L8G5 along with pcDNA3-*Zeb1* or pECE-*Foxo3* with 2 μl of Lipofectamine® 3000 (Thermo-Fisher Scientific) per well. Knockdown of gene expression with siRNAs was performed as described above. When cells needed to be transfected with both cDNA expression vectors and siRNAs, cells were first transfected with siRNAs (with RNAiMAX) for 5 h, washed and replaced with fresh medium. Twenty four hours later, cDNA expression vectors were transfected (Lipofectamine® 3000) during 5 h. As control, equal molar amounts of the corresponding expression empty vectors and/or siCtrl were also transfected. The total amount of transfected DNA was topped up to the same amount in all conditions with pBluescript SK. To normalize for transfection efficiency, 0.5 μg of SV40-β-galactosidase were co-transfected in each condition. Levels of luciferase and β-galactosidase activity were assayed 48 hours later with Luciferase Assay System kit (Promega Corp.) and Luminiscent β-galactosidase Detection kit II (Clontech, Takara), respectively. Relative luciferase activity (RLU) was determined using Modulus II Glomax microplate reader (Promega Corp.). Data shown correspond to the mean of at least three independent experiments with each transfection conducted as duplicates. When RLU values are represented in a histogram, the control condition is set to a RLU value of 100.

***In vivo* assessment of *Fbxo32* promoter transcription**

The gastrocnemius muscles of both hindlimbs of wild-type and *Zeb1* (+/-) mice were injected with 25 μg of the 3.5 kb *Fbxo32* promoter and 25 μl of a 10X (weight/volume) Pluronic L-64 (Sigma-Aldrich) diluted 1:2 in PBS as described (23). After 3.5 days, the left hindlimb was immobilized for 3.5 additional days. At day 7, luciferase activity was assessed by bioluminescent imaging (8). Briefly, mice were anesthetized with 500 mg/kg of Avertin (Sigma-Aldrich) and the plastic cast was removed. Then, mice were injected i.p. with 15 mM of CycLuc1 substrate (Calbiochem®, EMD Millipore, Billerica, MA) in 100 μl of PBS. Ten minutes later, the photon flux signal was collected in a charge-coupled ORCA-II BT imaging system (Hamamatsu Photonics, Hamamatsu City, Japan). Bioluminescence data was analyzed with Wasabi! Imaging Software (Hamamatsu Photonics) and represented as the total photon flux/sec/cm^2 signal emitted by each hindlimb using the signal emitted by the head and trunk as background reference.

Supplementary Table S1

siRNA oligonucleotides

Gene Target	Upstream sequence	Reference
siCtrl	UAUAGCUUAGUUCGUAACC	22
si*Zeb1-A*	GACCAGAACAGUGUUCCAUGUUUAA	22
si*Zeb1-B*	AACUGAACCUGUGGAUUAU	22
si*Ctbp*	GAACUGUGUCAACAAGGACTT	22
si*Foxo3*	Pool of 3 siRNAs [Santa Cruz Biotechnologies (sc-37888)]<br>CAUGCGCGUUCAGAAUGAAtt<br>GAACGUUGUUGGUUUGAAUtt<br>GAAGGAAGGUGUUAUAUCAtt	

Supplementary Table S2

Primary and secondary antibodies

Antibodies		
**Primary Abs**	**Source**	**Clone (Catalog Number)**
Atrogin-1	ECM Biosciences	AP2041
GAPDH	Cell Signaling	14C10
Laminin	Santa Cruz Biotechnologies	48H-2 (sc-59854)
MuRF1	Santa Cruz Biotechnologies	C-11 (sc-398608)
MYOD1	Santa Cruz Biotechnologies	G-1 (sc-377460)
ZEB1	Santa Cruz Biotechnologies	H-102 (sc-25388)
ZEB1	Santa Cruz Biotechnologies	E20-X (sc-10572)
ZEB1	Atlas Antibodies (Sigma-Aldrich)	HPA027524
**Secondary Abs**	**Source**	**Catalog Number**
Anti-rabbit Alexa Fluor 488	Jackson ImmunoResearch	711-545-152
Anti-rabbit -HRP	Jackson ImmunoResearch	111-035-144
Anti-mouse-HRP	Jackson ImmunoResearch	715-035-151
Anti-rat Rhodamine Red-X	Jackson ImmunoResearch	712-295-153

Supplementary Table S3

DNA primers used in qRT-PCR

Gene	Forward 5' – 3'	Reverse 5' – 3'	Reference
Ctsl	GTGGACTGTTCTCACGCTCAAG	TCCGTCCTTCGCTTCATAGG	13
4ebp1	CACGCTCTTCAGCACCAC	GGAGGCTCATCGCTGGTAG	This article
Fbxo32	GCAAACACTGCCACATTCTCTC	CTTGAGGGGAAAGTGAGACG	13
Foxo3	GATAAGGGCGACAGCAACAG	CTGTGCAGGGACAGGTTGT	15
Gabarapl1	CATCGTGGAGAAGGCTCTA	ATACAGCTGGCCCATGGTAG	13
Myod1	TGGGATATGGAGCTTCTATCGC	GGTGAGTCGAAACACGGATCAT	7
Nrf2	GCAACTCCAGAAGGAACAGG	AGGCATCTTGTTTGGGAATG	13
Psma1	CATTGGAATCGTTGGTAAAGAC	GTTCATCGGCTTTTTCTGC	This article
Gapdh	CGACTTCAACAGCAACTCCCACTCTTCC	TGGGTGGTCCAGGGTTTCTTACTCCTT	1
Trim63	TGTCTGGAGGTCGTTTCCG	ATGCCGGTCCATGATCACTT	5
Zeb1	AACTGCTGGCAAGACAAC	TTGCTGCAGAAATTCTTCCA	22

Supplementary Table S4

Oligonucleotides for site directed mutagenesis

Gene Target	Sequence
Upper strand	GAGCCTATAAACAAAGCCACTCAGCCTCGGGGCGCGGGGGG
Lower strand	CCCCCCGCGCCCCGAGGCTGAGTGGCTTTGTTTATAGGCTC

Supplementary Table S5

Oligonucleotides for ChIP assays

Promoter Region	Forward 5' → 3'	Reverse 5' → 3'
Fbxo32 promoter<br>ZEB1 binding site (-85 bp)<br>(-105/+48 bp)	GGCGAGCCTATAAACAAAGCCAC	CTCCTGACTCTGGGAATGCTGAG

REFERENCES FOR SUPPLEMENTARY MATERIAL AND METHODS

1. Banerjee, S., Xie, N., Cui, H., Tan, Z., Yang, S., Icyuz, M., Abraham, E. and Liu, G. (2013) MicroRNA let-7c regulates macrophage polarization. *J. Immunol.*, **190**, 6542-6549.

2. Blais, A., Tsikitis, M., Acosta-Alvear, D., Sharan, R., Kluger, Y., and Dynlacht, B.D. (2005) An initial blueprint for myogenic differentiation. *Genes & Dev*. **19**, 553-569.

3. Blau, H.M., Chiu, C.P., and Webster, C. (1983) Cytoplasmic activation of human nuclear genes in stable heterocaryons. *Cell* **32**, 1171-1180

4. Cai, D., Frantz, J.D., Tawa, Jr. N.E., Melendez, P.A., Oh, B.C., Lidov, H.G., Hasselgren, P.O., Frontera, W.R., Lee, J., Glass, D.J., and Shoelson, S.E. (2004) IKKβ/NF-κB activation causes severe muscle wasting in mice. *Cell* **119**, 285-298.

5. Castillero, E., Martín, A. I., López-Menduiña, M., Granado, M., Villanúa, M. Á., and López-Calderón, A. (2009). IGF-I system, atrogenes and myogenic regulatory factors in arthritis induced muscle wasting. *Mol. Cell. Endocrinol.*, **309**, 8-16.

6. Davis, R.L., Weintraub, H., and Lassar, A.B. (1987) Expression of a single transfected cDNA converts fibroblasts to myoblasts. *Cell* **51**, 987-1000.

7. Dogra, C., Changotra, H., Mohan, S., and Kumar, A. (2006). Tumor necrosis factor-like weak inducer of apoptosis inhibits skeletal myogenesis through sustained activation of nuclear factor-κB and degradation of MyoD protein. *J. Biol. Chem.*, **281**, 10327-10336.

8. Evans, M.S., Chaurette, J.P., Adams, Jr. S.T., Reddy, G.R., Paley, M.A., Aronin, N., Prescher, J.A., and Miller, S.C. (2014) A synthetic luciferin improves bioluminescence imaging in live mice. *Nat. Methods*, **11**, 393-395.

9. Jag, U.R., Zavadil, J., and Stanley, F. M. (2009). Insulin acts through FOXO3a to activate transcription of plasminogen activator inhibitor type 1. *Mol. Endocrinol*, **23**, 1587-1602.

10. Lemercier, C., Verdel, A., Galloo, B., Curtet, S., Brocard, M.P., and Khochbin, S. mHDA1/HDAC5 histone deacetylase interacts with and represses MEF2A transcriptional activity. *J. Biol. Chem.* **275**, 15594-15599.

11. Madaro, L., Smeriglio, P., Molinaro, M., and Bouché, M. (2008) Unilateral immobilization: a simple model of limb atrophy in mice. *Basic Appl. Myol.* **18**, 149-153.

12. Maurer, P., T'sas, F., Coutte. L., Callens, N., Brenner, C., Van Lint, C., De Launoit, Y., and Baert, J.L. (2003) FEV acts as a transcriptional repressor through its DNA-binding ETS domain and alanine-rich domain. *Oncogene.* **22**, 3319-3329

13. Milan, G., Romanello, V., Pescatore, F., Armani, A., Paik, J. H., Frasson, L., Seydel, A., Zhao, J., Abraham, R., Goldberg, A.L., Blaauw, B., DePinho, R.A., Sandri, M. (2015) Regulation of autophagy and the ubiquitin-proteasome system by the FoxO transcriptional network during muscle atrophy. *Nat Commun.* **6,** 6670.

14. Nakae, J., Cao, Y., Hakuno, F., Takemori, H., Kawano, Y., Sekioka, R., Abe. T., Kiyonari, H., Tanaka, T., Sakai, J., and Takahashi, S.I. (2012) Novel repressor regulates insulin sensitivity through interaction with Foxo1. *EMBO J.* **31**, 2275-2295.

15. Nowak, K., Killmer, K., Gessner, C., and Lutz, W. (2007). E2F-1 regulates expression of FOXO1 and FOXO3a. *Biochim Biophys Acta*, **1769**, 244-252.

16. Postigo, A., and Dean, D.C. (1999) Independent repressor domains in ZEB regulate muscle and T-cell differentiation. *Mol. Cell. Biol.*, **19**, 7961-7971.

17. Postigo A, and Dean DC. (1999) ZEB represses transcription through interaction with the corepressor CtBP. *Proc. Nat. Acad. Sci. U.S.A.*, **96**, 6683-6688

18. Sánchez-Tilló, E., de Barrios, O., Siles, L., Cuatrecasas, M., Castells, A., and Postigo, A. (2011). β-catenin/TCF4 complex induces the epithelial-to-mesenchymal transition (EMT)-activator ZEB1 to regulate tumor invasiveness. *Proc. Nat. Acad. Sci. U.S.A.*, **108**, 19204-19209.

19. Sandri, M., Sandri, C., Gilbert, A., Skurk, C., Calabria, E., Picard, A., Walsh, K., Schiaffino, S., Lecker, S.H., Goldberg, A.L. (2004) Foxo transcription factors induce the atrophy-related ubiquitin ligase atrogin-1 and cause skeletal muscle atrophy. *Cell* **117**, 399-412.

20. Sekido, R., Murai, K., Funahashi, J.I., Kamachi, Y., Fujisawa-Sehara, A., Nabeshima, Y.L., Kondoh, H. (1994) The δ-crystallin enhancer-binding protein δEF1 is a repressor of E2-box-mediated gene activation. *Mol Cell Biol.*, **14**, 5692-700.

21. Shirakihara, T., Saitoh, M., and Miyazono, K. (2007) Differential regulation of epithelial and mesenchymal markers by δEF1 proteins in epithelial–mesenchymal transition induced by TGF-β. *Mol. Biol. Cell*, **18**, 3533-44.

22. Siles, L., Sánchez-Tilló, E., Lim, J.W., Darling, D.S., Kroll, K.L, and Postigo, A. (2013) ZEB1 imposes a temporary stage-dependent inhibition of muscle gene expression and differentiation via CtBP-mediated transcriptional repression. *Mol Cell Biol.*, **33**, 1368-1382.

23. Song, H., Liu, S., Li, C., Geng, Y., Wang, G., and Gu, Z. (2014) Pluronic® l64-mediated stable hIF-1α expression in muscle for therapeutic angiogenesis in mouse hindlimb ischemia. *Intern J Nanomed.,* **9**, 3439-3452.

24. Takagi T, Moribe H, Kondoh H, Higashi Y. (1998) δEF1, a zinc finger and homeodomain transcription factor, is required for skeleton patterning in multiple lineages. *Development* **125**, 21-31.

25. Van Der Vos, K.E., Eliasson, P., Proikas-Cezanne, T., Vervoort, S.J., Van Boxtel, R., Putker, M., Van Zutphen, I.J., Mauthe, M., Zellmer, S., Pals, C., Verhagen, L.P. (2012) Modulation of glutamine metabolism by the PI(3)K-PKB-FOXO network regulates autophagy. *Nat. Cell Biol.,* **14**, 829-837.

Supplementary Figure S1

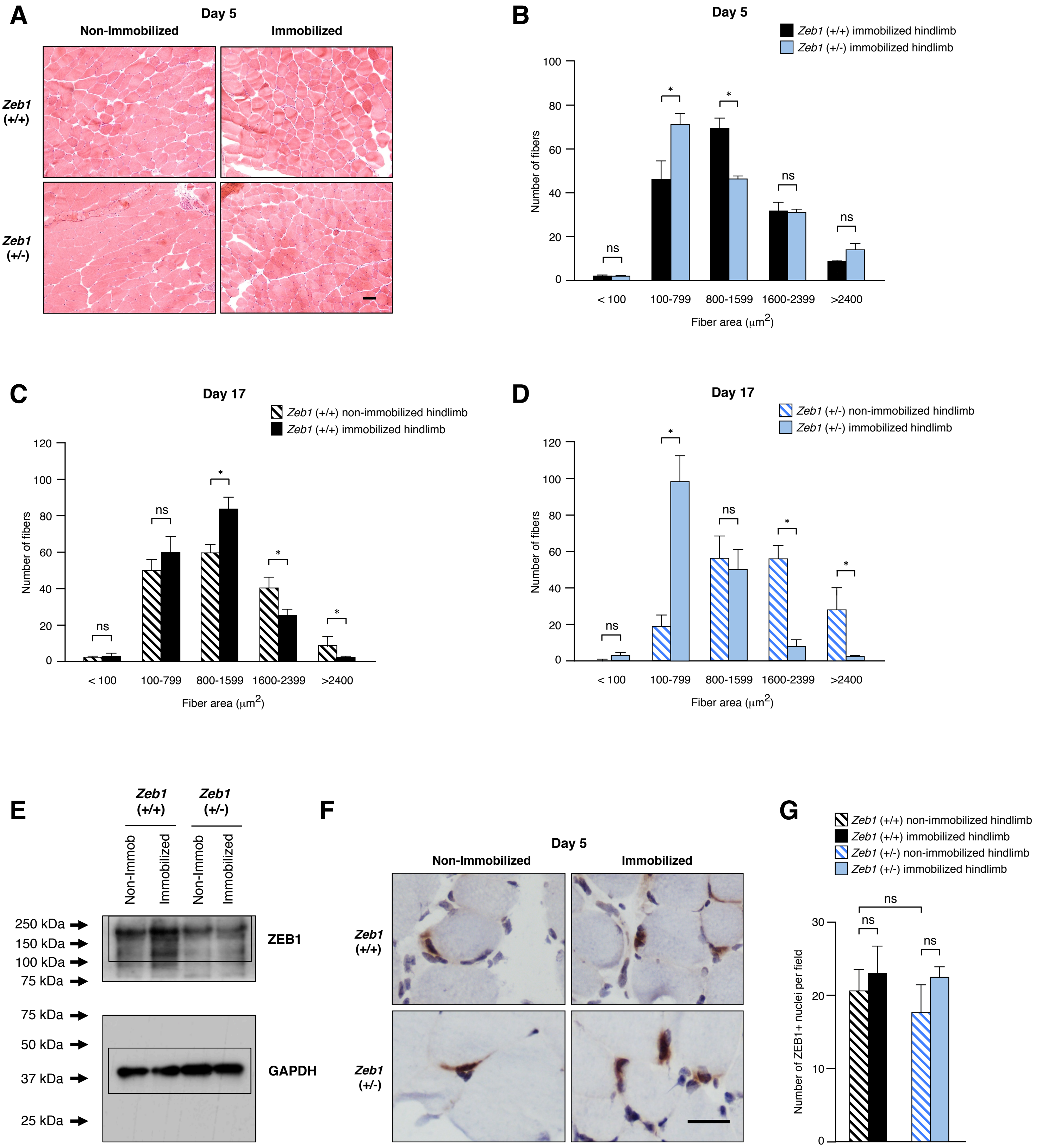

SUPPLEMENTARY FIGURE S1

***Zeb1* expression inhibits muscle sparing and myofiber reduction following immobilization.** **(A)** As in Figure 1C, but wild-type and *Zeb1* (+/-) mice were subjected to unilateral hindlimb immobilization during 5 days before euthanasia. Scale bar: 50 μm. **(B)** Distribution of the cross-sectional area of the myofibers in the gastrocnemius of the immobilized hindlimb from wild-type and *Zeb1* (+/-) mice at day 5 of the immobilization protocol. (**C)** As in (B) but comparing myofiber cross-sectional area of gastrocnemius in the non-immobilized (hatched bars) and immobilized (solid bars) hindlimbs of wild-type mice at day 17 of the immobilization protocol. The results are the mean with standard errors of at least five mice of each genotype. **(D)** As in (C) but in *Zeb1* (+/-) mice. **(E)** Full unedited blots for Figure 1G. As indicated in Supplementary Materials and Methods, Western blot membranes were split into two sections at the level of the 75 kDa protein molecular weight marker. The upper section was used to detect ZEB1 (clone HPA027524) while the lower section was used to detect GAPDH (clone 14C10). **(F)** Higher magnification of the captures shown in Figure 1H. **(G)** Immobilization does not alter the number of $ZEB1^{+}$ nuclei. As in Figure 1H, the absolute number of nuclei positive for ZEB1 per 20X magnification field was calculated in four fields. Data are the average of four mice for each genotype and condition.

Supplementary Figure S2

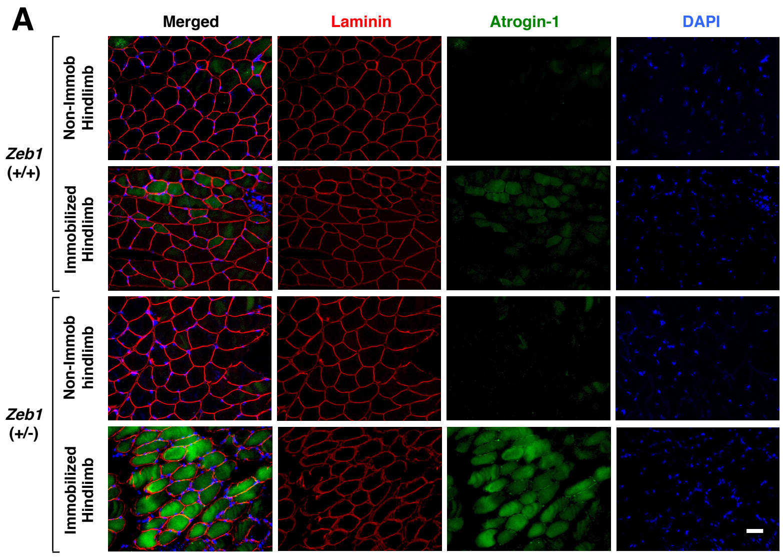

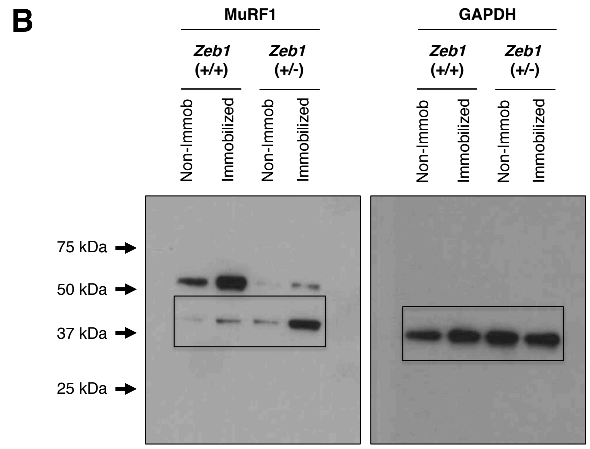

SUPPLEMENTARY FIGURE S2

***Zeb1* inhibits the *in vivo* induction of atrogenes upon immobilization. (A)** Single immunostaining corresponding to Figure 2B. Scale bar: 50 μm. **(B)** Full unedited Western blots corresponding to Figure 2D. As indicated in Supplementary Materials and Methods, Western blot membranes were split into two sections at the level of the 75 kDa protein molecular weight marker. The lower section was used to subsequently detect MuRF1 (clone C-11) and/or GAPDH (clone 14C10).

Supplementary Figure S3

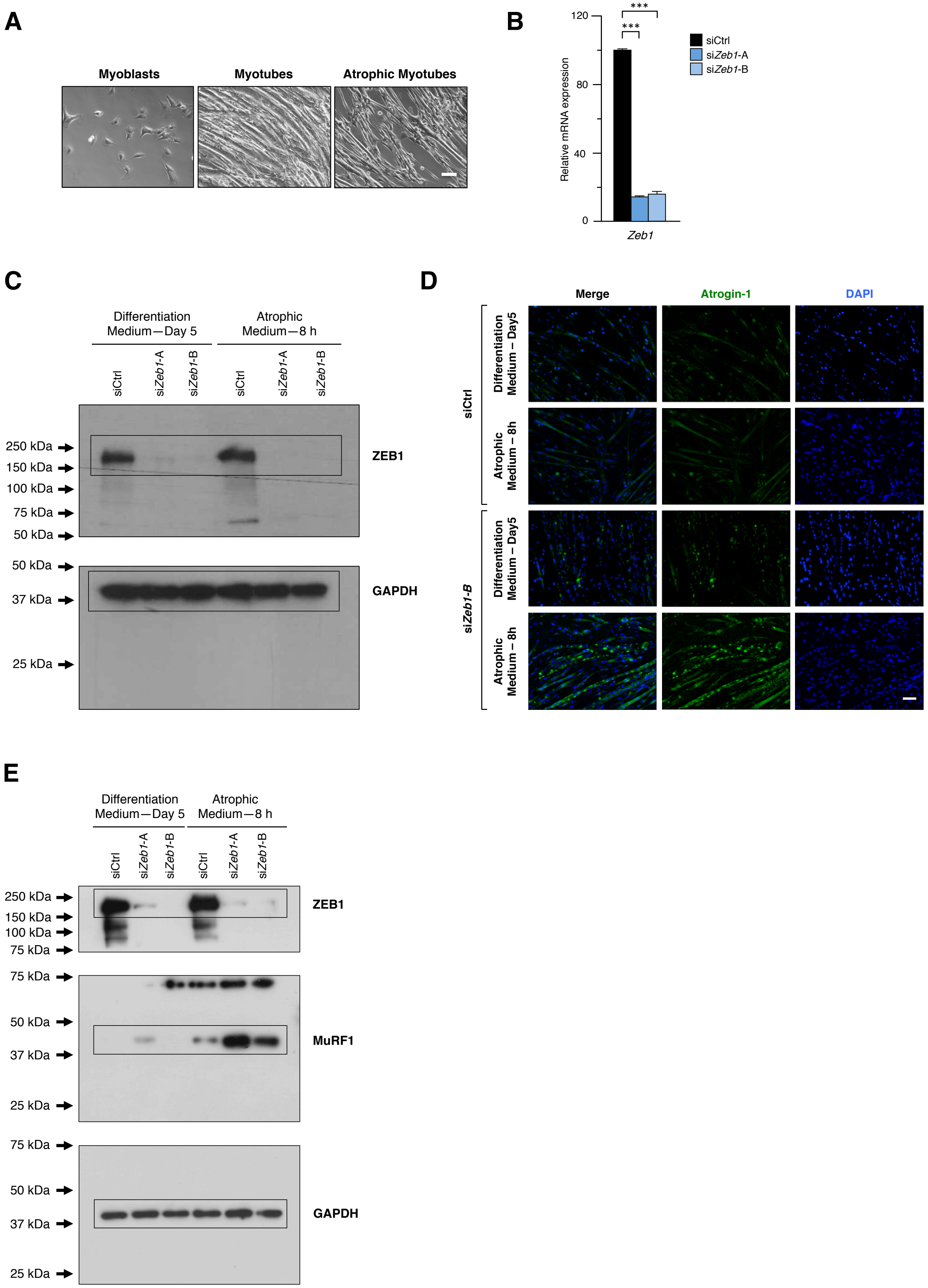

SUPPLEMENTARY FIGURE S3

ZEB1 inhibits atrogene expression and size reduction in starved C2C12 myotubes (A) When C2C12 cells are grown in growth medium, cells maintain a proliferating myoblast-like phenotype. Once they reach confluence and are cultured in differentiation medium they fuse to form terminally multinucleated myotubes (2,3). When C2C12 myotubes are cultured in atrophic medium, they undergo a reduction in myotube diameter (19). See Supplementary Materials and Methods for details. Scale bar: 50 μm. **(B)** C2C12 myotubes were transfected with a control siRNA (siCtrl) or with either of two previously validated siRNAs against *Zeb1* (si*Zeb1*-A and si*Zeb1*-B) (22). See Supplementary Materials and Methods for details. mRNA levels were assessed by qRT-PCR with respect to *Gapdh*. **(C)** Full unedited Western blots for Figure 3E. As indicated in Supplementary Materials and Methods, Western blot membranes were split into two sections. The upper section of the blot was used to detect ZEB1 (clone HPA027524) while the lower section was used to detect GAPDH (clone 14C10). **(D)** Single staining captures for Figure 3G. Scale bar: 50 μm. **(E)** Full unedited Western blots for Figure 3H. As in (C), but the membranes were split at the level of the of the 75 kDa protein molecular weight marker. The upper section of the blot was used to detect ZEB1 (clone HPA027524) while the lower section was used to subsequently detect MuRF1 (clone C-11), and GAPDH (clone 14C10).

Supplementary Figure S4

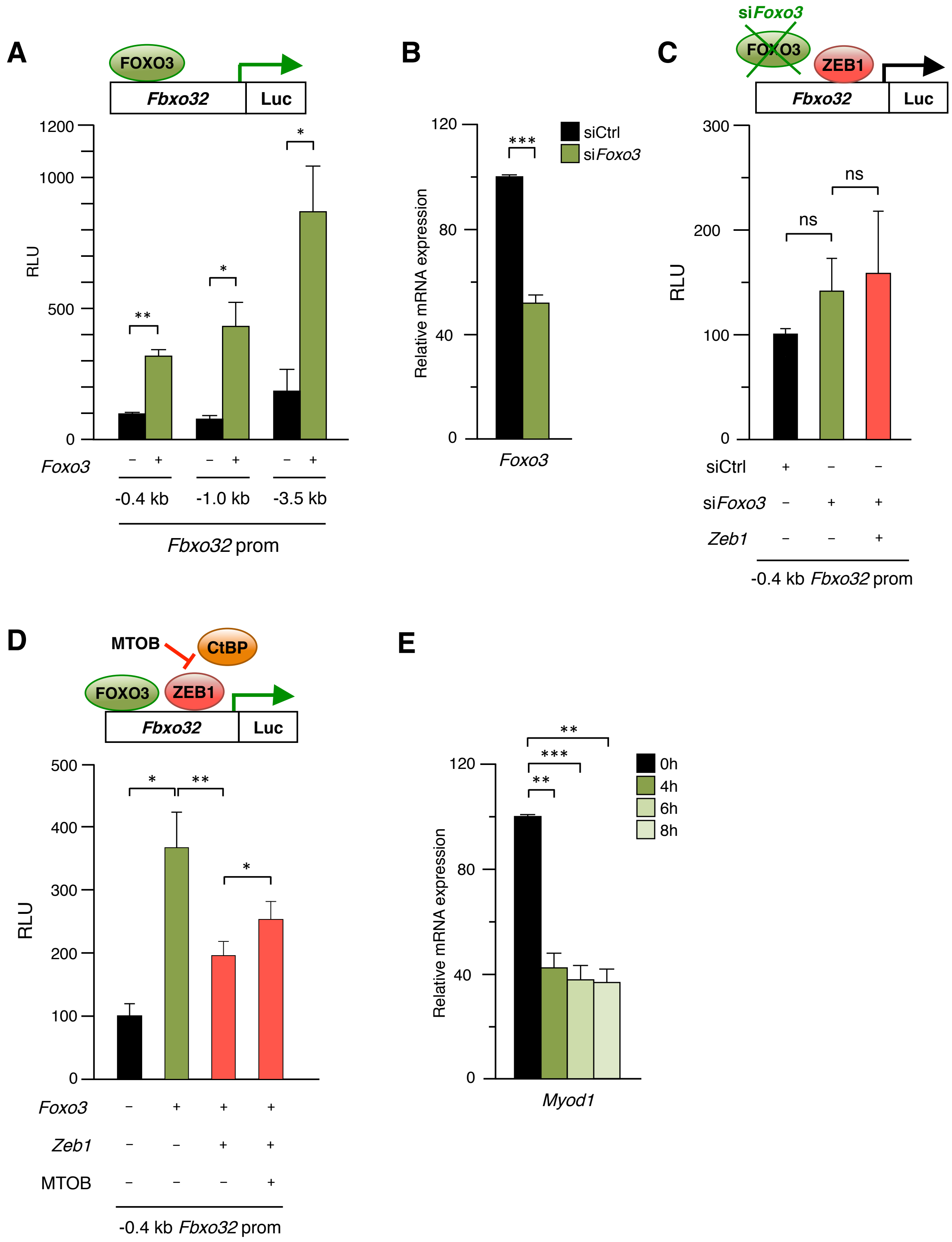

SUPPLEMENTARY FIGURE S4

ZEB1 represses the *Fbxo32* promoter through CtBP-dependent inhibition of FOXO3 transcriptional activity. (A) Transcriptional activity of different fragments of the *Fbxo32* promoter both under basal conditions and in response to *Foxo3* overexpression. C2C12 cells were transfected with equal molar amounts of luciferase reporters containing the 0.4 kb, 1.0, and 3.5 kb fragments of the mouse *Fbxo32* promoter (19) (0.43 μg, 0.48 μg, and 0.68 μg, respectively) along with 0.82 μg of *Foxo3* or equal molar amounts of the corresponding empty vector. **(B)** C2C12 cells were transfected with with 50-100 nM of either siCtrl or a pool of three siRNAs against *Foxo3* (si*Foxo3*). mRNA levels were assessed by qRT-PCR with respect to *Gapdh*. Data are the mean of four independent experiments. **(C)** C2C12 cells were transfected with 0.43 μg of a luciferase reporter containing a 0.4 kb fragment of the mouse *Fbxo32* promoter (19), 1.88 μg of an expression vector for *Zeb1* (or the corresponding molar amount of the empty expression vector) and/or 50-100 nM of either siCtrl or a pool of three siRNAs against *Foxo3* (si*Foxo3*). **(D)** C2C12 cells were transfected with 0.43 μg of a luciferase reporter containing a 0.4 kb fragment of the mouse *Fbxo32* promoter (19), 0.82 μg of an expression vector for *Foxo3* (or equal molar amounts of the empty vector), and/or 1.88 μg of an expression vector for *Zeb1* (or the corresponding molar amount of the empty vector), and in the presence or absence of 10 mM of MTOB. Data are the mean of three independent experiments. **(E)** *Myod1* expression is downregulated in atrophic myotubes. C2C12 myotubes were cultured in atrophic medium during 4, 6 and 8 h and *Myod1* expression was assessed by qRT-PCR with respect to *Gapdh*.
